# Odontological Society of England

**Published:** 1891-06

**Authors:** Henri Weiss

**Affiliations:** For Dr. J. Arkovy, of Buda-Pesth


### SELECTIONS.

### Odontological Society of England.—" Ready Contours and Crowns Made of Amalgam."

MR. HENRI WEISS—FOR DR. J. ARKOVY, OF BUDA-PESTH.

The best writers and practitioners had adopted contour filling. Gold was maintained to be the best material for the purpose, and no doubt it was in general; but taking into account the various conditions of approximal cavities in premolars and molars, which more frequently want contouring than front teeth, it might be conceded that contour fillings of gold were very rarely indicated in back teeth; first, because of the invariable absence of wall strength; secondly, the proximity of the cervix; thirdly, the impossibility of control while annealing the first quarter of the gold. To these arguments might be added the effective, and for the case valuable, indication of combined fillings, where gold had to be shelved as a noncombinable material in favor of amalgam. Providing, therefore, that amalgam could be used for shaping the lost part of the crown, apart from the saving of time, the foregoing reasons pointed to it as better than gold for contouring back teeth. These considerations had suggested to Dr. Arkovy the possibility of a ready made amalgam crown (or partial crown) being serviceable for the purpose. The premolars and molars which Dr. Arkovy had the pleasure of introducing to the profession, and which might be obtained of the dental depots, were made after natural teeth, and possess the precise morphological shape required for crown work, or, cut into segments, for contours. The directions for use were as follows:—(1.) Take impression, and bite from the prepared tooth, and selecting the anatomically suitable amalgam crown, have it fitted in the laboratory to the model, somewhat lower than articulation requires. If one part of the crown is wanted, have it cut by a thin saw, and filed as required, then let the inner part be provided with retaining pits or deep under-cuts. Time hardens, and air has no deteriorating effects upon the crowns. (2.) When starting the stopping, put the crown or contour into chloroform, in order to get rid of any fat that might possibly adhere. The amalgam for the filling being prepared, take a small quantity of it, to which add one small drop of mercury, and rub in with a polisher upon the surface furnished with under-cuts. Fill the amalgam into the cavity with or without rubber dam to the level of the edges, covering them as well. Put the amalgam crown, or contour, upon the filling, push the piece with the fore-fingers several times in the labio-lingual direction until it attains its proper position. No hard pressure should be exerted on the contours, their body being too weak, but entire crowns should be pressed firmly. Then polish, and if the rubber dam is in use remove it *very carefully*. In order to keep the contour or crown in position, a small piece of leather paper should now be introduced between it and the approximal surface of the next tooth. For entire upper crowns it is advisable to cautiously drill through the crown fissure, for the amalgam filling will more easily keep it in place and counteract its dropping before setting ensues. Special care should be taken to exclude moisture, to leave no space between the piece and tooth, and to save the work from unnecessary movement. After the lapse of four, preferably five, hours for setting, finishing may be effected with lava strips drawn through the interspace upon the cervical part. Polish with circular brush dipped first into alcohol and then in pumice stone powder. Patients should be dissuaded from masticating on that side for two or three days. Any preparation of amalgam may be used, but if copper amalgam, eight hours would be required for setting.

Dr. J. F. W. Silk, M.D., read a paper entitled :

"OBSERVATIONS ON BROMIDE OF ETHYL IN DENTAL SURGERY."

In America, on the Continent, and especially in Germany, the drug had been held in high repute by dental surgeons for some time past, but in this country there were no records of its systematic employment in a similar manner. At the outset Dr. Silk wished to disclaim any advocacy of the indiscriminate use of the bromide or any suggestion that it was an entirely satisfactory substitute for the other dental anæsthetics. He had endeavored to impartially record his table of cases, and he desired to be equally impartial in his remarks, so that each might form his own opinion as to whether the drug was worthy of a more extended trial. Bromide of ethyl seemed first to have been used as an anæsthetic by Nunneley, of Leeds, in 1849, and again brought forward by him in 1865. Some years later its use was advocated by Turnbull, and by Levis in America. For its introduction into dental work Dr. Silk believes Schneider, of Berlin, and Hertz, of Vienna, were responsible. As to its properties, without entering minutely into the subject, care should be taken that when bromide of ethyl ($C_2H_5Br.$) was asked for, the chemist did not supply bromide of ethylene ($C_2H_4Br.$), the substitution of which had led to fatal results. Merck's preparation was almost universally used in Germany, and notwithstanding a leading English chemist's assurance of the purity of some English preparations, Dr. Silk strongly favored Merck's. Variations in effect might be partly, or even entirely, due to the decomposition which the drug undergoes by keeping. As to the cost, where any number of administrations are undertaken, it might be said to be very little more than half the cost of nitrous oxide per patient. It would seem that robust and healthy males tolerate the drug better than weak anæmic females or children. The amount of the dose would vary *inter alia* with the method of administration, *e. g.*, in the first seven cases with the open or semi-open method 70 minims to 3 drachms were required. With the closed method 1½ drachms had usually proved ample, and in many instances 1 drachm had sufficed. He would be inclined to put the dose down at from 1 drachm to 1½ drachm. Being not altogether satisfied with the inhaler used in the first seven cases Dr. Silk adopted an ordinary Ormsby's inhaler, upon which the drug is poured through the face-piece on to the sponge and it is then fitted to the face; little or no air is admitted for the first few inhalations, after which the cap of the air-hole is slightly turned. The induction of anæsthesia with this inhaler averaged 66.8 seconds, and the duration 46.2 seconds—half as long again as with nitrous oxide. The average of induction was based upon the observation of twenty, and the average of duration upon twenty-eight cases.

Roughly, the duration of anæsthesia bears some relation to the time required for induction, but this is by no means absolute, and with the English preparations of the drug the variability is very marked. When the time from the commencement of the inhalation to the end of the anæsthesia exceeds two minutes the after-effects are likely to be bad. The question "How do you know when a proper degree of anæsthesia has been produced?" is not so simply answered as might seem. In Berlin some stress seemed to be laid upon the muscular relaxation, but Dr. Silk had not found this at all reliable. He generally removed the face-piece when the faintest stertor was heard, or if that were obviously due to accidental causes, or unduly delayed, the commencing weakness of the pulse or the development of conjunctival insensibility were his guides. The stertor referred to is not the laryngeal stertor of nitrous oxide narcosis, but of a much lighter and regular kind originating probably in the palate. During the course of the inhalation it would probably be found that the breathing becomes slightly slower and shallower, but in the majority of cases these changes are almost inappreciable. Dr. Silk had never been able to detect any irregularity in the rhythm, nor, as a general rule, had any cough, laryngeal spasm, or bronchial irritation been set up. These remarks apply only to the methods of dental anæsthesia, and were not applicable to the effects produced when the anæsthesia is prolonged for longer periods. There could be no doubt, in his opinion, that the cardio-vascular, or circulatory system is much disturbed during the inhalation of bromide of ethyl. The primary flushing of the face is, he believed, a pretty constant phenomenon, though it might be but momentary in its duration. This effect is very similar to that produced by nitrite of amyl, and might possibly be explained in the same way, *i. e.*, arterial dilatation with consequent fall of blood pressure. This fall might, no doubt, to a certain extent, in itself account for the diminution in force and increased frequency of the heart-beat as observed at the radial pulse. It was, however, highly probable that other factors are at work for the flushing quickly succeeds a pallor, and the heart's action tends to become more feeble, slower and irregular.

Dr. Silk thought upon the whole that his sphymographic tracings (exhibited) bore out the views he had put forward in respect to the effects of the bromide on the circulatory system, especially if it be remembered that the heart's action immediately before the inhalation is probably unduly accelerated by the nervous condition of the patient. One set of tracings he regarded as particularly interesting as indicating the rapidity with which the blood pressure, etc., is restored. This rapid recovery is, he thought, usual and is of great importance.

As to the effect of the drug upon the nervous system, and through it upon the muscular system, the exceedingly transient character of the stage of excitement was striking, in the majority of cases it had been almost inappreciable. This observaton seemed to apply almost equally to the nervous woman and the robust. It might possibly serve as an indication for the class of cases in which the use of the drug is more particularly appropriate. Occasionally very slight spasm of the fingers, or rhythmic movement of the limbs, etc. were exhibited, but Dr. Silk had never observed any thing like the degree of spasm and movement which often accompanies nitrous oxide or ether. In respect to other phenomena he had not much to say. The pupils tend to dilate. Increase in the salivary secretions was sufficiently marked in four cases to attract attention, but never so copious as with ether. In some few instances patients would make a groaning noise from the beginning of the inhalation, but this had always been ignored and did not either delay or shorten the narcosis. The anæsthesia which had resulted from the use of the drug had, to Dr. Silk's mind, always been quite satisfactory both in degree and in kind.

After-effects might be divided into immediate and remote. Of the former Dr. Silk had definite notes in thirty-nine instances. In eleven a certain amount of hysteria resulted, in ten others more or less depression or prostration occurred. In one actual fainting ensued, but the patient was subject to what she termed "fainting fits." Actual sickness was noted in two cases, and in seven others nausea was complained of. Slight and transient degrees of exhilaration were developed in three instances, and in many cases a certain amount of confusion of the intellect had remained for some few minutes. By the use of printed post-cards rough estimates of remote effects had been obtained in forty-five instances. Of these fory-five, in no fewer than twenty-nine, the return was to the effect that the patients were perfectly well on their return home and for the remainder of the day. Prostration or depression was noted in five instances, vomiting in two and headache in three. In one case the return was, "ill all day," but the nature of the illness Dr. Silk was not able to ascertain. Possibly it might have been simple hysteria. Of the remaining five cases dizziness, drowsiness, and other very trivial and doubtful complaints were recorded. As to the relation between immediate and remote effects, from seventeen tabulated observations of both, it was gathered that the importance and severity of the remote symptoms were tolerably proportionate with the immediate after-effects. Thus simple hysteria had seldom been followed by any thing worse, while on the other hand, nausea might be followed by sickness, or the sickness might be prolonged. This somewhat negative evidence was of importance for it enabled Dr. Silk to say, so far as his own limited experience was concerned, that he had never met with a case in which serious symptoms had developed upwards of twelve hours after the bromide had been inhaled. Such cases had, however, been described. With reference to the death-rate and dangers of bromide of ethyl, while aware of the importance of the point, he could not help thinking that the fatalities which had been hitherto recorded rather tended to exaggerate the danger of the drug when used in dental surgery in the way he had described.—*Dental Record.*

---

"THERE is law and order pervading every part of the jaw, a wise design being apparent in the arrangement of the teeth and jaws in consonance not only with the laws of geometry, physics and mechanics, but according to the most approved methods of modern science for the economy of force."

ROBT. M. SOLOMONS, D.D.S., Charleston, S. C.

